# *In-situ* nano-crystal-to-crystal transformation synthesis of energetic materials based on three 5,5′-azotetrazolate Cr(III) salts

**DOI:** 10.1038/srep37587

**Published:** 2016-11-21

**Authors:** Yu Miao, Yanxuan Qiu, Jiawei Cai, Zizhou Wang, Xinwei Yu, Wen Dong

**Affiliations:** 1 Department of Chemistry, Guangzhou Key Laboratory for Environmentally Functional Materials and Technology, Guangzhou University, Guangzhou 510006, P.R. China

## Abstract

The *in-situ* nano-crystal-to-crystal transformation (SCCT) synthesis provides a powerful approach for tailoring controllable feature shapes and sizes of nano crystals. In this work, three nitrogen-rich energetic nano-crystals based on 5,5′-azotetrazolate(AZT^2−^) Cr(III) salts were synthesized by means of SCCT methodology. SEM and TEM analyses show that the energetic nano-crystals feature a composition- and structure-dependent together with size-dependent thermal stability. Moreover, nano-scale decomposition products can be obtained above 500 °C, providing a new method for preparing metallic oxide nano materials.

Both the synthetic methodology and morphology of size and shape control are very important in the practical application for nano materials^1^,^2^,^3^,^4^,^5^,^6^,^7^,^8^,^9^,^10^. However, the previous synthetic approaches for nano energetic materials exhibited limited control over the shape and size distribution of these particles because they are dangerous explosives^11^,^12^,^13^. It is currently a prerequisite urgency and an extremely challenging task to develop the mild and universal construction methods for the further investigation of nano energetic materials^14^,^15^,^16^,^17^,^18^. Currently, the controllable preparation of nano energetic materials with distinct structures and feature sizes is of great importance to achieve desirable properties, such as structure- and size-dependent properties, including energy density, thermal decomposition, sensitivity and operational performance^18^,^19^,^20^,^21^,^22^. Up to now, although some successful synthetic methods such as re-precipitation^23^, rapid expansion of supercritical solutions^24^ and physical vapor deposition (PVD) techniques^25^ have been extensively used for the fabrication of nano energetic materials, it is still very difficult to reduce the size of the nano energetic materials attributing to their dangerous explosive properties^11^,^12^,^13^. In the past decades, the high-energy density materials (HEDMs) based on AZT^2−^ organic and inorganic salts have been widely synthesized and found practical applications^12^,^26^,^27^,^28^. For example, Tl and Pb metal salts (Tl_2_AZT and Pb_2_(OH)_2_AZT) had been used as initiators for an extremely high nitrogen content (84.3%) of H_2_AZT, and their detonation parameters can be compared to 1,3,5-trinitroperhydro-1,3,5-triazine (RDX) and the majority of its decomposition products is N_2_, showing excellent environmental benign^12^,^26^,^27^,^28^. In our previous work, the single crystal structure for *cis* isomer of AZT^2−^ anion was obtained in a Ni(II) complex of [Ni(2,2′-bpy)_3_]_2_(*cis*-AZT)(*trans*-AZT)·10H_2_O]^29^. To the best of our knowledge, the detailed investigations of nano energetic materials based on AZT^2−^ metal salts remain unexplored although nano-scale arrays can increase energy density, insensitivity and performance^4^. Up to now, the SCCT synthetic method has not been used to prepare the energetic nano-crystal although such preparation methodology gave an useful enlightenment for the design and fabrication of the target energetic nano-crystal and tailoring unique nano structures, feature shapes and sizes. In regard to the above mentioned considerations, three nitrogen-rich energetic nano-crystals salts based on AZT^2−^ and Cr(III), formulated as [Cr(H_2_O)_6_]_2_(AZT)_3_·6H_2_O (**1**), [Cr(OH)(AZT)(H_2_O)_3_]·2H_2_O (**2**) and [Cr(OH)(H_2_O)_5_]AZT·H_2_O(**3**), were synthesized by the simple and controllable SCCT synthetic method. Their decomposed products and morphology-dependent thermal properties were characterized and demonstrated.

## Results

**Figure 1:**
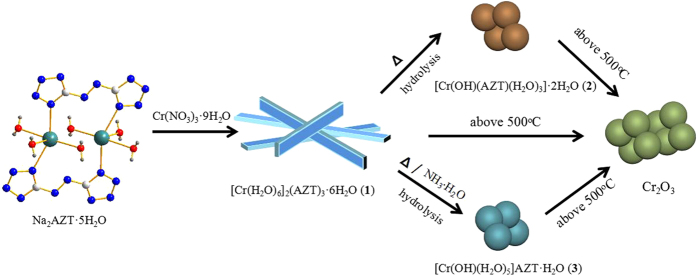
Preparation of 1–3 and transformation progress from 1 to 2 and 3 and their decomposed products above 500 °C.

**Figure 2:**
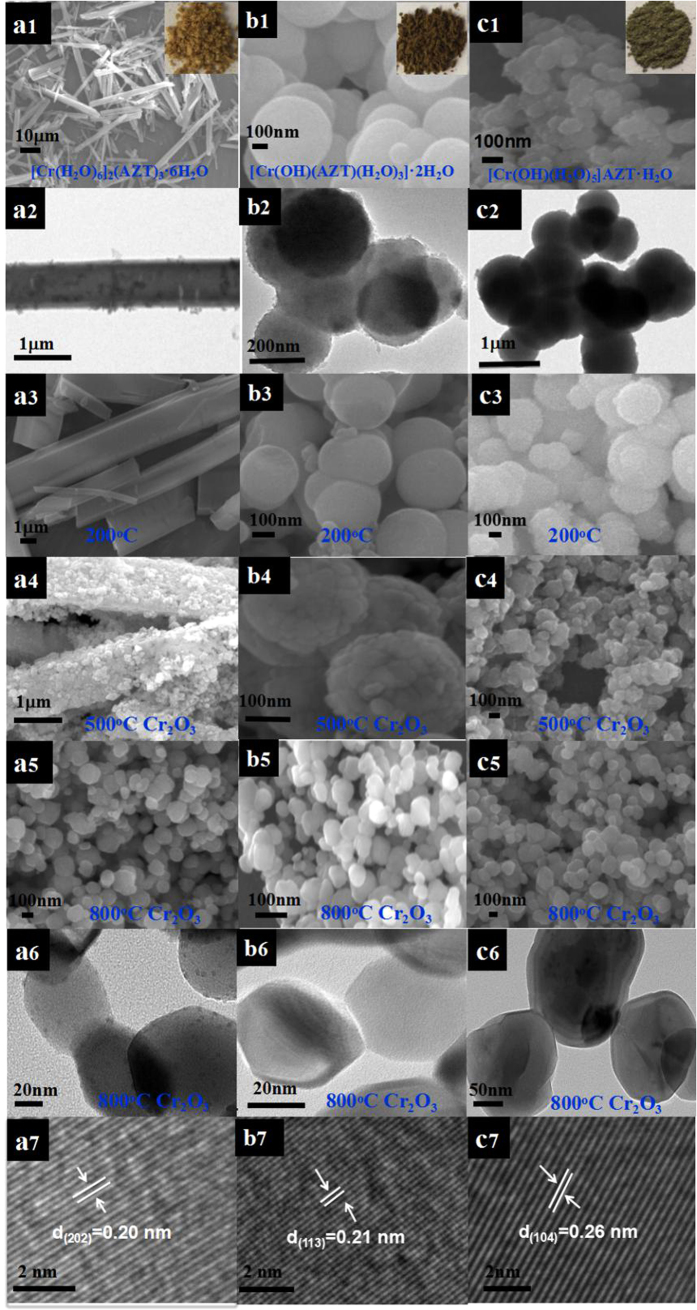
SEM and TEM images of nano crystals of **1** (a1, a2), **2** (b1, b2) and **3** (c1, c2) and their nano-scale dehydrated and decomposed products (a3–a6, b3–b6, c3–c6). High-resolution TEM images showing different lattice of crystals of **1**–**3** (a7, b7, c7).

The chromium nitrate [Cr(NO_3_)_3_·9H_2_O] and disodium 5,5′-azotetrazolatesalt of [Na_2_(AZT)·5H_2_O] were selected to prepare the targeted energetic nano-crystals of **1**–**3**. The synthetic strategy (Fig. 1) and SEM, TEM images for the energetic nano-crystals of **1**–**3** and their *in-situ* decomposed products are shown in Fig. 2.

### Syntheses and SEM, TEM images for nano crystals of 1–3 and their *in-situ* decomposed products at 200, 500 and 800 °C

This new synthetic method is based on a controllable SCCT approach by a heating hydrolysis and thermolysis strategy. Briefly, the chromium nitrate and disodium azotetrazolate salts were firstly dissolved in water with lower concentration control, respectively. Then the two kinds of solutions were mixed and stirred at ambient conditions and terminated within 20–30 minutes. Rapid precipitation resulted in hexagonal prism-shaped yellow nano rods with the thickness of 200–500 nm, observed by scanning electron microscopy (SEM) (Fig. 2a1) and transmission electron microscopy (TEM) (Fig. 2a2). Then the original mixture of **1** was heated to about 70 °C for 30 minutes, the hexagonal prism-shaped nano rods were *in-situ* transformed to extremely small orange-yellow nano spherical particles with an average diameter ranging from 100 to 200 nm observed by SEM (Fig. 2b1) and TEM (Fig. 2b2). If the reaction temperature and time are lowered to 70 °C over 30 minutes, some of the hexagonal prism-shaped nano rods can still be observed rods, indicating that not all the rods could be completely transformed (Fig. S1). When 2–4 drops of dilute NH_3_·H_2_O (2 mol·dm^−3^) were added dropwise to the original mixture of **1** in order to change the pH of the solution and then the new mixture was further heated to 70 °C for 30 minutes under stirred condition, the hexagonal prism-shaped nano rods were *in-situ* transformed to another new green nano spherical particles of **3** with an average diameter ranging from 200 to 500 nm observed by SEM (Fig. 2c1) and TEM (Fig. 2c2). When the nano crystals of **1**–**3** with ca. 10 mg were placed in dry pot and calcined for 2 hours at 200, 500 and 800 °C, respectively, the original nano crystals were dehydrated and *in-situ* decomposed to a series of new nano intermediates at 200 °C (Fig. 1a3, b3, c3) and finally transformed to Cr_2_O_3_ nano metallic oxide crystals above 500 °C with uniform size distribution of average size ca. 50–100 nm observed by SEM and TEM (Fig. 2a3–a6, b3–b6, c3–c6). As noticed, the dehydrated and decomposed nano intermediate at 200 °C can basically keep the original shape and size of **1**–**3** (Fig. 2a3, b3, c3). The high resolution TEM images (Fig. 2a7, b7, c7) for the decomposed nano crystals at 800 °C reveal that the distances of 0.22, 0.21 and 0.26 nm should be correspond to the 202, 113 and 104 planes of Cr_2_O_3_ nano particles, respectively (PDF No. 38–1479).

The chemical compositions of **1**–**3** with the proposed structural formulas of [Cr(H_2_O)_6_]_2_(AZT)_3_·6H_2_O (**1**), [Cr(OH)(AZT)(H_2_O)_3_]·2H_2_O (**2**) and [Cr(OH)(H_2_O)_5_]AZT·H_2_O (**3**) were determined by elemental analysis (EA), powder X-ray diffraction (PXRD), Raman and IR spectra (Supplementary Fig. S2–S4).

### The XPS spectra of three nano crystals of 1–3 and their decomposed products at 200 and 500 °C are shown in Fig. 3

**Figure 3:**
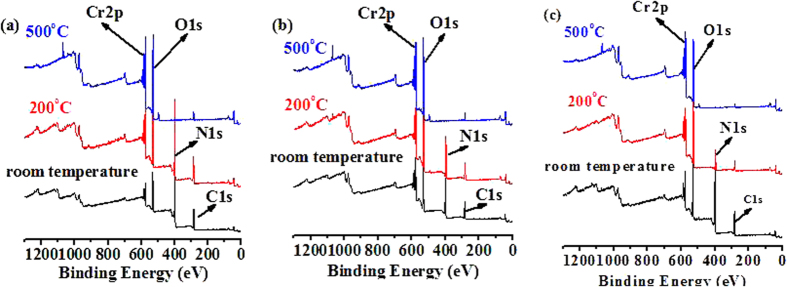
XPS for nano crystals of **1**–**3** (**a, b, c**) and their decomposed products at 200 and 500 °C.

The high resolution XPS spectra of **1** in Fig. 3a demonstrates that the Cr_2p_ and O_1s_ characteristic peaks of the decomposed products at 200 and 500 °C are observed at 577.08 and 531.08 eV, respectively. Two characteristic peaks for C_1s_ at 285.08 eV and N_1s_ at 401.08 eV are noticed in **1** and its decomposed products at 200 °C, indicating that the nano crystal of **1** has been decomposed to intermediate under this temperature. The N_1s_ characteristic peak is not noticed but a weak C characteristic peak originating from the foreign substance is still observed in the decomposed products at 500 °C, which indicates that the nano crystal of **1** has been completely decomposed and transformed to Cr_2_O_3_ nano products above 500 °C. Both **2** (Fig. 3b) and **3** (Fig. 3c) show similar XPS spectroscopic properties to that of **1**. The XPS spectra further confirm the chemical compositions of **1**–**3** and their decomposed products.

### Thermogravimetric analysis (TG) differential scanning calorimetry (DSC) are performed here to resolve the thermal stability and decomposition mechanism for nano crystals of 1–3

**Figure 4:**
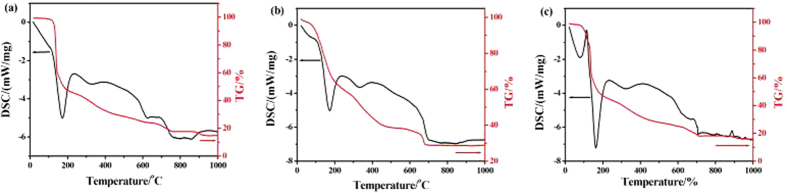
TG and DSC analysis for micro/nano crystals 1–3.

In order to evaluate the thermal stability of the synthesized nano crystals of **1**–**3**, TG–DSC and DTG experiments were performed under N_2_ atmosphere. The TG–DSC results for the samples **1**–**3** (heated up to 1000 °C at a rate of 10 °C/min in an argon flow) are shown in Fig. 4. There’s no distinct steps shown in the TG–DSC curves for **1** (Fig. 4a). The nano rod of **1** begins to lose the lattice water till 103 °C, which indicates that the crystal water molecules are stable maybe due to their stronger intermolecular hydrogen bonds. The rapid weight loss immediately occurs and reaches to 35% at 138 °C corresponding to theoretical mass loss of 35.2% for eighteen lattice and coordinated water molecules in **1**. Three DTG peaks are observed at 132, 349 and 458 °C, respectively (Fig. S5a). However, no endothermic peak corresponding to the loss of water molecules is observed in the DSC curve, which is ascribed to the exothermic decomposition follows immediately at the temperature that coordinated water begins to be released (Fig. 4a). TG analysis further confirms the proposed chemical composition of **1** with formulation of Cr_2_(AZT)_3_·12H_2_O. After dehydration, the nitrogen content of the residual increases to 82.55%, implying the enhancement of energy. However, the transformation of crystal structure throws light on the instability of the dehydration product and the exothermic decomposition follows immediately and an exothermic peak is observed at 171.9 °C in the DSC curve (Fig. 4a) with the amount of heat release of 935 J/g. The weight loss reaches 51.5% at 210 °C, which indicates that the nano crystal of **1** has been decomposed to an intermediate of 1/2[Cr_2_(AZT)_3_]·Cr_2_O_3_ at this temperature. From 210 to 999 °C, the residual of **1** is continually decomposed with three small exothermic peaks being further noticed at 322, 620 and 761 °C and finally combusts to Cr_2_O_3_ nano product with residual weight of 14.7% at 999 °C, which is lower than the theoretical value of 16.5%.

Similarly, the TG–DSC curve for **2** also shows no distinct steps (Fig. 4b). The sample of **2** begins to lose the crystal water molecules at room temperature, which indicates that the crystal water molecules are instable maybe due to a weak intermolecular hydrogen bonds. The weight loss reaches 11.1% at 114.0 °C, corresponding to the weight loss of two crystal water molecules in **1** with the residual formula of [Cr(OH)(AZT)(H_2_O)_3_]. Then the weight loss reaches 27.8% at 164.0 °C, which corresponds to the weight loss of all crystal and coordinating water molecules with the residual formula of [Cr(OH)(AZT)]. After dehydration, the exothermic decomposition follows immediately and no endothermic peak corresponding to the loss of water molecules is noticed in the DSC curve. Three DTG peaks for rapid weight loss are observed at 86, 117, 169 °C, respectively (Fig. S5b). The weight loss reaches 39.5% at 238.0 °C, indicating that the nano crystal of **2** has been decomposed to an intermediate of 1/2[Cr(OH)(AZT)·Cr_2_O_3_] under this temperature and an exothermic peak is observed at 172.7 °C in the DSC curve (Fig. 4b) with the amount of heat release of 887.7 J/g. From 208 to 999 °C, the residual of **2** is continually decomposed and two small exothermic peaks were further noticed around 331 and 761 °C in the DSC curve. Finally, the residual of **2** combusts to Cr_2_O_3_ nano products with residual weight of 28.96% at 999 °C, which is higher than the theoretical value of 24.28%.

The sample of **3** begins to lose the crystal water at 78 °C (Fig. 4c). Then, the rapid weight loss immediately reaches 36.71% with the residual formula of [Cr(OH)]AZT at 136 °C with an endothermic peak corresponding to the loss of all water molecules being noticed at 95 °C with 229 J/g amount of absorption of heat. Two DTG peaks are observed at 93, 127 °C (Fig. S5c), respectively. After dehydration, the exothermic decomposition follows immediately and the rapid weight loss reaches 48.1% with the residual formula of 1/2[Cr(OH)(AZT)·Cr_2_O_3_] at 177 °C and an exothermic peak is observed at 162 °C in the DSC curve (Fig. 4c) with the amount of heat release of 683.4 J/g. After continually heated to 999 °C, the residual of **3** are further decomposed and combusts to Cr_2_O_3_ nano product with residual weight of 15.84% at 999 °C, which is lower than the theoretical value of 22.22%. Three exothermic peaks are further observed around 331, 656 and 706 °C in the DSC curve. The dehydrated and decomposed phenomenon indicates that the samples **1–3** are impossible to dry at higher temperature. However, based on above SEM imaging and XPS spectra analysis, the nano rod crystals of **1**–**3** can keep the original shape and size at 200 °C, indicating a composition- and structure-dependent together with morphology-dependent thermal stability.

### The sensitivity characteristics for nano crystals of 1–3

The results of friction and impact sensitivity are showed in Table 1 by using the previous reported method^30^. It shows that the impact sensitivity of **1** compared to RDX is lower than octahydro tetranitro tetrazocine (HMX) and its friction sensitivity is lower than RDX and HMX. The impact sensitivity of **2** and **3** are higher than RDX and HMX and their friction sensitivity compared to RDX are lower than HMX, implying that **1**–**3** can act as initiators. The hexagonal prism-shaped nano rod of **1** has higher impact sensitivity and lower friction sensitivity than those of spherical nano particles of **2** and **3**, implying a morphology-dependent impact and friction sensitivity because of the fact that both **2** and **3** have different surface area than that of **1**. The flame sensitivity is characterized by the height corresponding to 50% fire probability (H_50_). The lower H_50_ values imply the higher thermal sensitivity of **1**–**3**. The very lower values for electrostatic sensitivity test show that **1**–**3** are very insensitive to electrostatic ignition. The above results show that **1**–**3** may be used as potential initiators or detonators.

## Discussion

A simple and controllable SCCT synthetic method was applied to fabricate the energetic nano-crystals of AZT^2−^ Cr(III) salts. The morphology for the targeted energetic nano-crystals was modulated by means of by heating hydrolysis and therefore a rod, and two spherical nano particles of **1**–**3** were successfully synthesized. This is the first report about fabrication of nano energetic crystals using the simple and controllable synthetic method. These nano energetic crystals of **1**–**3** show different composition- and structure-dependent together with morphology-dependent thermal stability and sensitivity and the formation of nano-scale decomposition products above 500 ^o^C provide a novel preparation method for metallic oxide nano crystals. Due to the controllable crystalline phase and special morphology for the nano products, the current strategy has far-reaching implications in the designing and preparation of numerous inorganic-organic hybrid energetic materials and substrates with special shapes, and thereby serving as a potential foreground in various areas especially in practical applications.

## Materials and Methods

### Caution

The complexes of disodium 5,5′-azotetrazolatesalt in its deprotonated anions are potentially explosive and should be handled in small quantities, especially when these compounds are synthesized under heating condition.

All commercial reagents and solvents were used without further purification unless otherwise stated. The disodium azotetrazolate salt 0.0420 g (0.2 mmol) was firstly dissolved in 10 ml water, and then 5 ml aqueous solution of the chromium nitrate 0.0800 g (0.2 mmol) was poured into. The mixed solution was stirred at ambient conditions for 20 minutes, then the yellow crystals of complex **1** were obtained. The original mixture of **1** was heated to about 70 °C for 20 minutes, small nano crystals of complex **2** were observed. The aqueous solution of the disodium azotetrazolate salt 0.1050 g (0.5 mmol) and the chromium nitrate 0.2000 g (0.5 mmol) was stirred at ambient conditions for 5 minutes. Then the mixture was heated to about 70 °C, when the colour of the mixture was turned to dark green, 3 drops of dilute NH_3_·H_2_O (2 mol·dm^−3^) were added into the original mixture in order to change the pH of the solution under stirred condition, and the new mixture was further heated to 70 ^o^C for 20 minutes, then the nano powders of complex **3** were obtained.

SEM images were performed using JSM-7001F field emission scanning electron microscope. TEM images were carried out using Tecnai F20 field emission transmission electron microscopy. IR spectra were recorded as pressed KBr pellets on a Bruker Tensor 27 spectrophotometer with an average of 64 scans. Raman spectra were obtained from the solid phase, using Bruker VERTEX 70 Raman instrument. Elemental analyses were carried out using a Perkin Elmer analyzer model 240. DTG analyses were carried out using the NETZSCH TG209F3 thermogravimetric analyzer. TG and DSC measurementswere performed using a TADSCQ10 calorimeter equipped with an autocool accessoryand calibrated using indium (heating up to 1000 °C at a heating rate of 10 °C/min in argon flow). XPS analyses were performed on an X-ray Photoelectron Spectroscopy/ESCA (ESCALAB 250, Thermo Fisher Scientific) which includes a monochromatized Al Kα X-ray source, a hemispherical analyzer, and a position sensitive detector. Powder x-ray diffraction (PXRD) intensities for **1**–**3** were measured at room temperature using a Bruker D8 X-ray diffractometer (Cu-Kα, λ = 1.54056 Å) and the crushed poly-crystalline powder samples were prepared by crushing the nano crystals and scanned from 5–60° with a step rate of 0.1° s^−1^. Friction, impact, flame and electrostatic sensitivity were measured in accordance with national military standard method GJB772A-97 602.1, GJB772A-97 601.1, GJB5891.25-2006 and WJ9038.3-2004 respectively. The testing conditions of friction sensitivity were that the weight of rocking hammer was 1.5 kg, the switch angle was 90^o^, the pressure was 3.92 MPa, and the quantity of sample was 20 mg. Twenty-five samples were tested and the firing percent was calculated. The testing conditions of impact sensitivity were that the weight of dropping hammer was10 kg, the height of dropping hammer was 25 cm, and the quantity of sample was 50 mg. Twenty-five samples were tested and the firing percent was calculated. The testing conditions of flame sensitivity were that the quantity of sample was 20 mg, and the samples were accumulated naturally and not flat. Thirty samples were tested and the the height corresponding to 50% fire probability (H_50_) was got. The testing conditions of electrostatic sensitivity were that discharge voltage was 7 kV, the discharge capacitance was 0.22 μF, the polarity of discharge was negative, the series resistance was 50 kΩ, the discharge gap was 0.50 mm, and the quantity of sample was 20 mg. Thirty samples were tested and the firing percent was calculated. The results are showed in Table 1.

## Additional Information

**How to cite this article**: Miao, Y. *et al.* *In-situ* nano-crystal-to-crystal transformation synthesis of energetic materials based on three 5,5′-azotetrazolate Cr(III) salts. *Sci. Rep.* **6**, 37587; doi: 10.1038/srep37587 (2016).

**Publisher’s note**: Springer Nature remains neutral with regard to jurisdictional claims in published maps and institutional affiliations.

## Supplementary Material

Supplementary Information

## Figures and Tables

**Table 1:** The sensitivity characteristics for nano crystals of 1–3.

Samples	Impact sensitivity (%)	Friction sensitivity (%)	Flame Sensitivity (cm)	Electrostatic sensitivity (%)
**1**	78 (64–88)	24 (13–38)	<1	0 (0, 0)
**2**	56 (41–70)	96 (87–100)	3.1	0 (0, 0)
**3**	12 (4–25)	90 (78–97)	<1	0 (0, 7)
RDX	72–78	88		
HMX	100	100		
