# Supplementary material for: In-situ nano-crystal-to-crystal transformation synthesis of energetic materials based on three 5,5′-azotetrazolate Cr(III) salts
Source: Sci Rep. 2016 Nov 21;6:37587. doi: 10.1038/srep37587 (PMC5116643; doi:10.1038/srep37587)
Supplement: Supplementary Information [file srep37587-s1.doc]

***In-situ* nano-crystal-to-crystal transformation synthesis of energetic materials based on three 5,5'-azotetrazolate Cr(III) salts**

Yu Miao^1^, Yanxuan Qiu^1^, Jiawei Cai^1^, Zizhou Wang^1^, Xinwei Yu^1^ ,and Wen Dong^1^,*

^1^Department of Chemistry, Guangzhou Key Laboratory for Environmentally Functional Materials and Technology, Guangzhou University, Guangzhou 510006, P.R. China.

These authors contributed equally to this work. *e-mail: dw320@aliyun.com


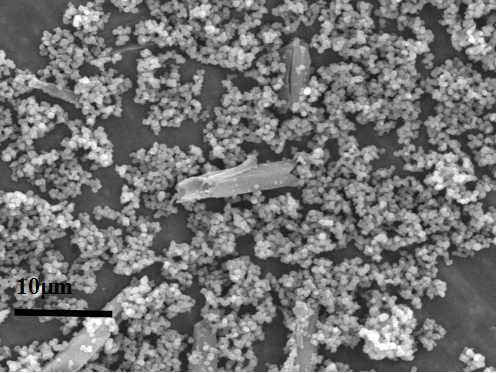


**Supplementary Figure S1.** Hexagonal prism-shaped nano rods could not be incompletely transformed and were still observed.

The C, H and N element content experimental values (%) of 1 are 8.06, 3.62 and 45.97, respectively, thus the empirical formula of **1** could be proposed as Cr_2_(AZT)_3_·12H_2_O in which the calculated element content values (%) for C, H and N are 7.83, 3.91 and 45.65, respectively. The experimental and theoretical values agree well with each other. Similarly, the C, H and N element content experimental values (%) of **2** are 7.80, 3.59 and 43.07, respectively. Based on the elemental analysis the empirical formula of **2** could be proposed as Cr(OH)(AZT)·5H_2_O in which the corresponding element content theoretical values (%) for C, H and N are 7.43, 3.40 and 43.34, respectively. Both the experimental and theoretical values agree well with each other. Based on the elemental experimental analysis values (%) of 7.16, 3.78 and 41.00 for C, H and N, respectively, the empirical formula of **3** could be proposed as Cr(OH)AZT·6H_2_O in which the corresponding element content theoretical values (%) for C, H and N are 7.04, 3.81 and 41.05, respectively. Both the experimental and theoretical values agree well with each other.

Three nano crystals of **1–3** were characterized by powder X-ray diffraction (PXRD) measurement (Fig. S2). The main characteristic diffraction peaks for three samples are sharp and basically coincident with each other, which indicate that they have the excellent crystallinity and similar main compositions. Because the standard JCPDS cards corresponding three samples of 1–3 have not been found, the three nano crystals should belong to new substances.


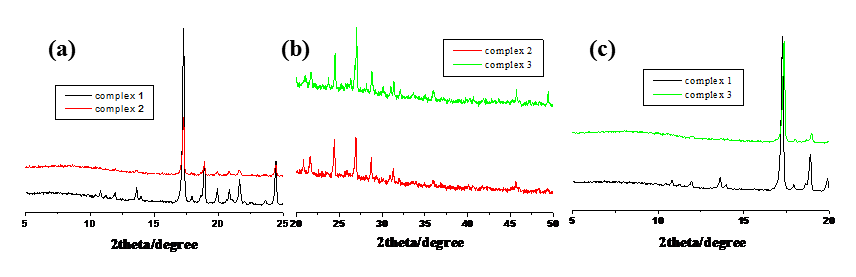


**Supplementary Figure S2.** Powder X-ray diffraction (PXRD) patterns for **1**–**3**.

Intense signals for AZT anion in the Raman spectra of three products of **1**–**3** have been observed (Fig. S3a). The characteristic peaks for two samples of **1** and **3** are very consistent with around 1485 cm^–1^ for azo stretching, 1381 cm^–1^ corresponding to the sym-metric C–N azo stretching, and 1081cm^-1^ corresponding to the tetrazole stretching being observed^1^. The appearance of these characteristic bands implies that the AZT^2–^ dianions act as counter ions and show non-coordinating bond with Cr(III) ion and this result supports the proposed structural formulas of [Cr(H_2_O)_6_]_2_(AZT)_3_·6H_2_O (**1**) and [Cr(OH)(H_2_O)_5_]AZT·H_2_O (**3**)^2,3^. However, the sample of **2** exhibits four main characteristic bands at 1067, 1257, 1229 and 1158 cm^–1^ that are different those of **1** and **3**, which indicates that the AZT^2–^ dianion in **2** should be a coordinating bond with Cr(III) ion. This result supports the proposed structural formula of [Cr(OH)(AZT)(H_2_O)_3_]·2H_2_O for **2**.

**Supplementary Figure S3.** Raman (a) and IR (b) spectra of **1**–**3**.

The IR spectra (Fig. S3b) of the three samples of **1**–**3** show an unresolved broadband around 3050 cm^–1^ for **1** and **3**, 3350 cm^–1^ for **2** corresponding to ν(O–H) stretching. The low value reveals that the OH group is involved in intermolecular O–H…N hydrogen bonding with AZT^2–^ dianion in which the interaction in **1** and **3** are more stronger than that of **2** due to its AZT^2–^ dianions in **2** being a coordinating bonding environment^3^. The characteristic bands around 1408 cm^–1^ for the asymmetric C–N_3_ stretching and 739 cm^–1^ for the asymmetric C–N_2_ stretching of the AZT^2–^ dianion were noticed^2^. The two characteristic bands further confirm the existence of the AZT dianion^2,3^. The IR spectra (Fig. S4) for the nano decomposed products can provide insight into their compositions. Two distinct sharp bands around 3357 and 1561 cm^–1^ were observed in their decomposed products at 200 ^o^C, which should be assigned to ν_as_(N–H) stretching and ν_as_(C–N) stretching. The characteristic bands around 1408 and 739 cm^–1^ for the AZT^2–^ dianion were not observed, which indicate that AZT^2–^ dianion have been decomposed to intermediate at 200 ^o^C. However, in IR spectra for their decomposed products at 500 and 800 ^o^C (Fig. S4), only a sharp characteristic band around 540 cm^–1^ for ν(Cr–O) stretching was noticed. This result indicates that the AZT^2–^ anions have been completely decomposed and in situ transformed to Cr_2_O_3_ products above 500 ^o^C.


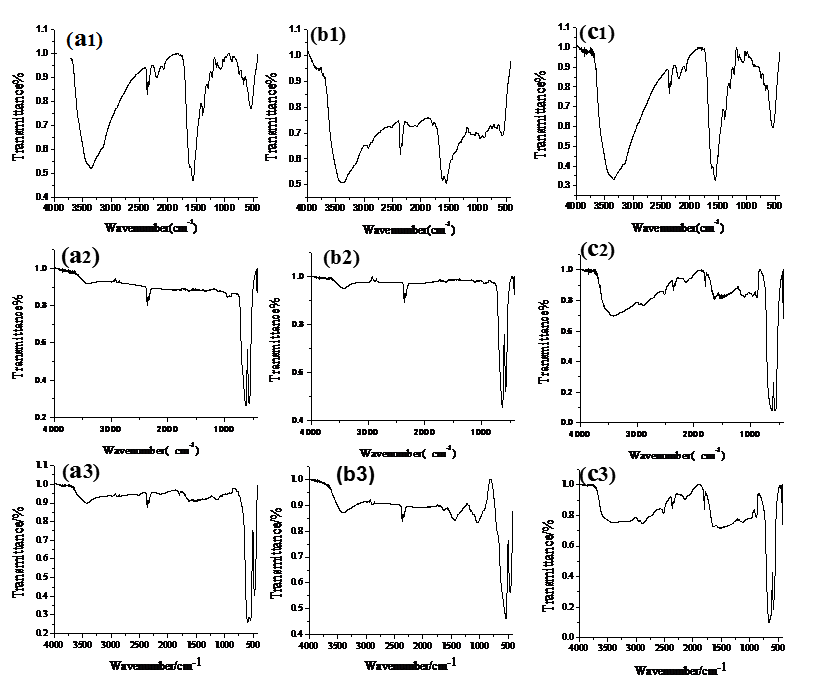


**Supplementary Figure S4.** IR spectra of nano-scale decomposition products (a1–a3, b1–b3 , c1–c3). a1–c1,a2–c2 and a3–c3 are the decomposition products of 1–3 at 200, 500 and 800 ^o^C.


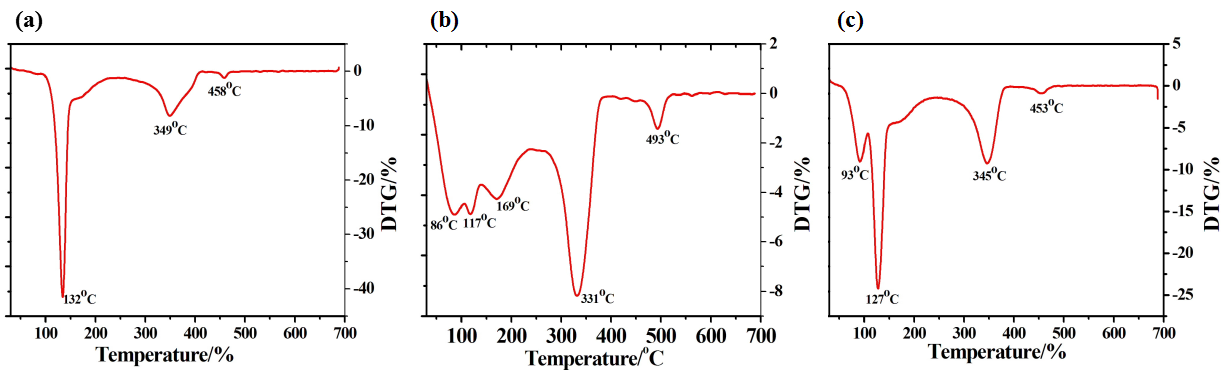


**Supplementary Figure S5.** DTG analysis for micro/nano crystals of **1**–**3**.

**References**

1. Jürgen Evers, *et al*. Cocrystallization of photosensitive energetic copper(II) perchlorate complexes with the

nitrogen-rich ligand 1,2-Di(1H-tetrazol-5-yl)ethane. *Inorg. Chem.* **53**, 11749−11756 (2014).

2. Lin, J. M. *et al*. Syntheses, structures and multi-photoluminescence images with confocal microscopy for three

5,5’-azotetrazolate(AZT) based Zn(II) and Ni(II) complexes. *Chem. Commun.* **47**, 2402–2404 (2011**)**.

3. Hammerl, A. *et al*. Salts of 5,5′-azotetrazolate. *Eur. J. Inorg. Chem.* **4**, 834–845 (2002).
